# Variation and distribution in temporalis muscle innervation: A systematic review and meta-analysis of anatomical studies

**DOI:** 10.1016/j.jpra.2024.10.018

**Published:** 2024-11-01

**Authors:** Zhen Yu Wong, Kai Qi Ou, Koen J.A.O. Ingels, Niels van Heerbeek, Sjaak Pouwels

**Affiliations:** a United Lincolnshire Hospitals NHS Trust, United Kingdom; b Department of Otorhinolaryngology and Head & Neck Surgery, Radboudumc, Nijmegen, The Netherlands; c Department of Surgery, Marien Hospital Herne, University Hospital of Ruhr University Bochum, Herne, NRW, Germany; d Department of Intensive Care Medicine, Elisabeth-Tweesteden Hospital, Tilburg, The Netherlands

**Keywords:** Temporalis, Innervation, Meta-analysis

## Abstract

**Background:**

Despite extensive discourse on the utilisation of the temporal muscle for facial reanimation, anatomical description regarding the innervation of its motor nerve branches is incomplete and varied. This systematic review aimed to consolidate the existing evidence concerning the distribution and variation in the pattern of temporalis innervation.

**Methods:**

A PRISMA-compliant systematic literature search was conducted in November 2023 and included studies offering anatomical insights into the distribution and variation of temporalis innervation patterns. Multiple category prevalence and proportional meta-analysis were conducted.

**Results:**

The initial search yielded 978 results, from which 13 studies were selected for inclusion. The inferior anterior temporalis muscle region was found to receive innervation from the buccal nerve's superior branch and inferior posterior region from the masseteric nerve. In the superior part, comprising anterior, middle and posterior regions, innervation is provided by the branches of the deep temporal nerve (DTN) arising from various branches of the mandibular or masseteric nerves. Analysis revealed that the most common variation of DTN was the presence of two branches (46%, 95% CI: 46%-63%, I^2^=94%), followed by three branches (26%, 95% CI: 24%-39%). Subgroup analyses of 86 patients indicated high prevalence rates of innervation by the temporal branches of the buccal nerve (85%, 95% CI: 76%-92%, I^2^=98%) and temporal branches of the masseteric nerve (72%, 95% CI: 63%-81%, I^2^=90%).

**Conclusion:**

In conclusion, this systematic review and meta-analysis highlight the evolving understanding and complexities of temporalis innervation, revealing the variations in nerve branches and emphasising the need for further research.

## Introduction

Since the onset of the 20th century, the temporalis muscle (TM) has emerged as an important element in the realm of facial reconstructive surgery, particularly in the context of dynamic facial reconstructions for individuals with peripheral facial palsy.^1^ Its significance spans a wide array of surgical interventions, showcasing its versatility and utility in addressing various facial impairments. The pioneering work of Lexer and Rosenthal in the early 1900s marked the inception of using a segment of TM to activate the orbicularis oris muscle after facial paralysis.^2^ Building upon this foundation, Gillies, in 1933, introduced the concept of facial reanimation through lengthening temporalis myoplasty.^3^ This innovative technique involved the reflection of the central portion of the TM over the zygomatic arch, complemented by the incorporation of a loop of the fascia lata to extend its reach to the nasolabial fold. A comparable principle was elaborated by Labbé, where the origin and insertion of TM were detached and the coronoid tendon was reinserted into the nasolabial fold, leaving the vascular and neurogenic pedicle intact. The TM flap has become indispensable in the spectrum of procedures encompassing the reconstruction of oral defects, cranial base reconstruction, facial reanimation surgery, midface augmentation and obliteration of orbital defects. The deep temporal nerve (DTN) has also emerged as a potential donor nerve for the reconstruction of facial nerve branches.^4^

Most textbooks on human anatomy delineate the TM as being innervated by the deep temporal branch of the anterior trunk of the mandibular nerve. Despite the extensive discourse on the utilisation of the TM for facial reanimation (where innervation of the TM should remain intact), anatomical description of the innervation of its motor nerve branches remains incomplete and varied. Moreover, the extent of variation in innervation between the left and right faces or in branching patterns remains insufficiently explored. Enhancing the understanding of temporalis innervation holds promise for devising more intricate designs for muscle or nerve transfer in future clinical management. Therefore, this systematic review aimed to consolidate the existing evidence concerning the distribution and variation in the pattern of TM innervation.

## Methods

### Information source

The objective of this systematic review was to identify qualitative studies conducted in the English language. To ensure a rigorous synthesis process, we adhered to the guidelines provided by the Cochrane Qualitative & Implementation Methods Group^5^ and evidence-based anatomy.^6^ Our search strategy involved comprehensive searches of the MEDLINE and EMBASE databases from their inception until November 14, 2023, along with manual screening of references and relevant literature. Specific search terms included in our study focused on the variations in TM innervation. Our analysis encompassed primary data and literature-based studies examining this topic.

### Study selection and eligibility criteria

Following the importation of the search results into the Mendeley reference manager, two independent reviewers (ZYW and KQO) evaluated the titles and abstracts of the retrieved articles to determine their potential relevance for the comprehensive review focused on the anatomical variation of TM innervation. Inclusion criteria were limited to original, peer-reviewed, full-length published articles written in English or translated into English. Any discrepancies during the full-text review between the reviewers were resolved through discussion and consensus. This research specifically aimed to investigate the studies examining the distribution and variations in innervation patterns of the TM.

### Data extraction and analysis

Author KQO conducted the data collection process, ensuring the reliability and accuracy of the collected information. Author ZYW performed a comprehensive review to further enhance the quality of the data. Relevant data points were extracted for each study included in the review. These encompassed descriptive attributes such as author, year of publication and country of study. Furthermore, additional factors extracted from each study consisted of the numbers and types of specimens used and distribution or variations related to TM innervation.

### Quality assessment

To assess the quality of the included studies, two reviewers (KQO and ZYW) conducted independent evaluations. The QUality Appraisal for Cadaveric Studies (QUACS) scale was used as a tool for this purpose.^7^ During a consensus meeting, the reviewers cross-validated their assessments to ensure consistency and accuracy. Based on the evaluation, the studies were categorised using the specific criteria outlined in a table. Studies that scored 1 to 6 out of 13 questions as ‘yes’ were classified as poor quality, studies scoring 7 to 9 were categorised as moderate quality and studies scoring 10 to 13 were considered as good quality (Table 1).

**Table 1:** Risk of bias assessment.

Study*	1	2	3	4	5	6	7	8	9	10	11	12	13	Total score#
Akita 2001^12^	1	1	0	0	0	0	1	0	0	0	1	1	0	5
Ali 2012^1^	1	0	1	0	0	0	1	0	0	0	1	1	1	7
Burggasser 2002^11^	1	0	1	0	0	0	1	0	0	1	1	1	0	7
Chang 2013^10^	1	1	1	0	0	0	1	0	0	1	1	0	0	6
Dauwe 2016^20^	1	1	1	0	0	0	1	0	0	1	1	0	0	6
Geers 2005^16^	1	0	0	0	1	0	1	1	1	1	1	1	0	8
Hwang 2004^17^	1	1	1	0	0	0	1	1	0	1	1	0	0	7
Karagoz 2015^19^	1	1	0	0	0	0	1	0	0	1	1	0	0	5
Nakajima 1998^13^	1	1	1	1	1	0	1	0	1	1	1	1	1	11
Pouwels 2022^20^	1	0	0	0	1	0	1	0	1	1	1	1	0	7
Shimokawa 1998^14^	1	1	1	1	1	1	1	1	0	1	1	1	0	11
Wong 2022^18^	1	0	1	0	1	0	1	0	0	1	1	1	0	7
Ziccardi 1998^15^	1	0	1	1	1	0	1	1	0	1	1	1	0	9

* Items of the QUACS scale: 1. Objective stated; 2. Basic information about the sample is included; 3. Applied methods are described comprehensively; 4. Study reports condition of the examined specimens; 5. Education of the dissecting researchers is stated; 6. Findings are observed by more than one researcher; 7. Results presented thoroughly and precisely; 8. Statistical methods are appropriate; 9. Details about consistency of the findings are given; 10. Photographs of the observations are included; 11. Study is discussed within the context of the current evidence; 12. Clinical implications of the results are discussed; 13. Limitations of the study are addressed.^7^

# Range; Low quality: 0-6; Moderate quality: 7-9; High quality: 10-13

### Statistical analysis

To perform this meta-analysis, MetaXL version 5.3 software (EpiGear International Pty Ltd., Wilston, Queensland, Australia), was used.^8^ Multiple category prevalence and proportional meta-analysis were conducted. A fixed effects model was used. The Chi-squared test and I-squared statistic were chosen to assess the heterogeneity among the studies. The p-values and confidence intervals were used to determine the statistical significance between the studies. A P-value ≤ 0.05 was considered statistically significant. The differences were considered statistically insignificant in the event of overlapping confidence intervals. I-squared statistics were interpreted as follows: values of 0–40% were considered as ‘might not be important’, values of 30–60% were considered as ‘might indicate moderate heterogeneity’, values of 50–90% were considered as ‘may indicate substantial heterogeneity’ and values of 75–100% were considered as ‘may indicate substantial heterogeneity’.

## Results

**Figure 1:**
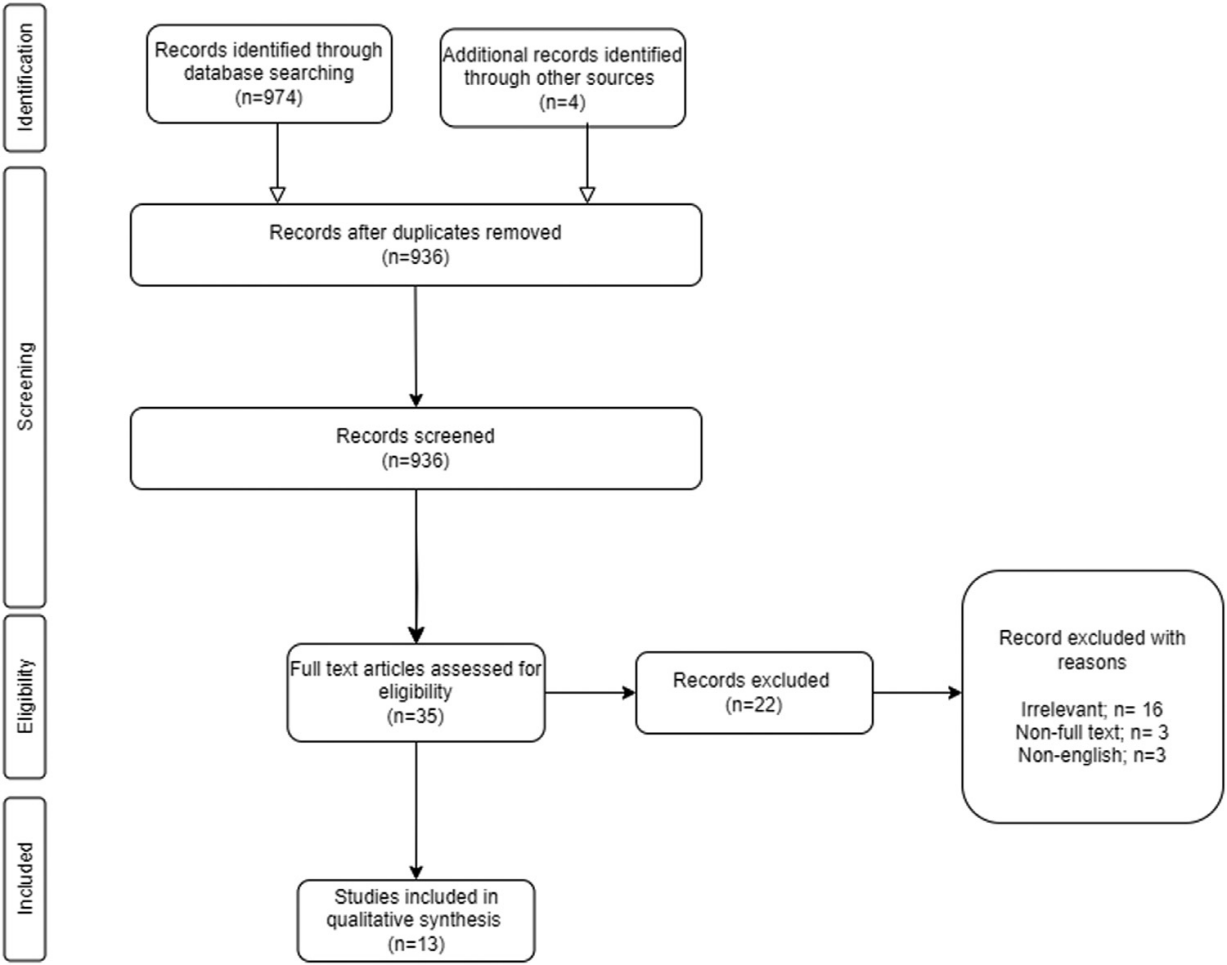
PRISMA diagram.

The primary literature search yielded a total of 974 results and 4 additional studies were identified through other sources; among these 42 were identified as duplicates. Following screening based on title and abstract, 35 studies were initially included and subsequently subjected to a full-text critical appraisal, resulting in the exclusion of 22 studies. Ultimately, a total of 13 studies were deemed eligible for inclusion in this systematic review.^1^^,^^4^^,^^9^, ^10^, ^11^, ^12^, ^13^, ^14^, ^15^, ^16^, ^17^, ^18^, ^19^ The details of the screening process are summarised in Figure 1. The aggregated data from these studies encompassed a total of 173 cadavers, with 3 studies conducted in Japan, 2 studies in the United States, 1 study in Austria, 1 study in Belgium, 1 study in the United Kingdom, 1 study in Singapore, 1 study in Netherlands, 1 study in Turkey and 1 study in Canada (Table 2). The quality assessment results are presented in Table 1. Among the 13 included studies, 4 were classified as poor quality, 7 as moderate quality and only 2 studies met the criteria for good quality. Seven studies investigated the distribution of TM innervation and 7 studies investigated its variation, with one study reporting both outcomes. Some studies were dedicated to analysing the distribution of nerves that innervate the distinct anatomical regions within TM, focusing on delineating the differences in nerve distribution across these areas. Concurrently, other studies explored the variations in the dissimilarities in the DTN, temporal branch of the buccal nerve and temporal branch of the masseteric nerve which innervated TM.

**Table 2:** Summary of the included studies.

Study	Country	Number of cadavers (male /female)	Number of dissected temporalis muscles (unilateral/bilateral)	Distribution	Variation
Akita 2001^12^	Japan	34 (18/16)	66 (0/33)	YES	
Ali 2012^1^	United Kingdom	11 (5/6)	17 (5/6)		YES
Burggasser 2002^11^	Austria	60 (27/33)	43 (43/0)	YES	YES
Chang 2013^10^	Canada	10 (10/0)	10 (10/0)	YES	
Dauwe 2016^20^	United States of America	16 (7/9)	30 (30/0)		YES
Geers 2005^16^	Belgium	10 (x/x)	20 (0/10)	YES	
Hwang 2004^17^	Korea	16 (11/5)	16 (16/0)		YES
Karagoz 2015^19^	Turkey	5 (2/3)	10 (0/5)		YES
Nakajima 1998^13^	Japan	1 (x/x)	1 (1/0)	YES	
Pouwels 2022^20^	Netherlands	5 (3/2)	10 (0/5)		YES
Shimokawa 1998^14^	Japan	5 (3/2)	10 (0/5)	YES	
Wong 2022^18^	Singapore	18 (x/x)	18 (18/0)		YES
Ziccardi 1998^15^	United States	8 (x/x)	8 (8/0)	YES	

### Distribution of TM innervation

Anterior-posterior relation of TM innervation was investigated in 5 studies. Shimokawa et al. and Burggasser et al.^10^^,^^13^ suggested that the TM could be partitioned into three parts: anterior, middle and posterior. Geers et al. reported that the front part received its nerve supply from the DTN or buccal nerve.^15^ Shimokawa et al. and Akita et al. found that it was specifically the anterior DTN that provided innervation.^11^^,^^13^ Burggasser et al. observed that the anterior region is supplied exclusively by the DTN, whereas the posterior region is innervated by either the posterior DTN alone or by a combination of the posterior DTN, deep temporal nerve and masseteric nerve.^11^ A deeper and denser innervation was noted in the anterior part of TM compared to the posterior part.^12^^,^^14^

**Figure 2:**
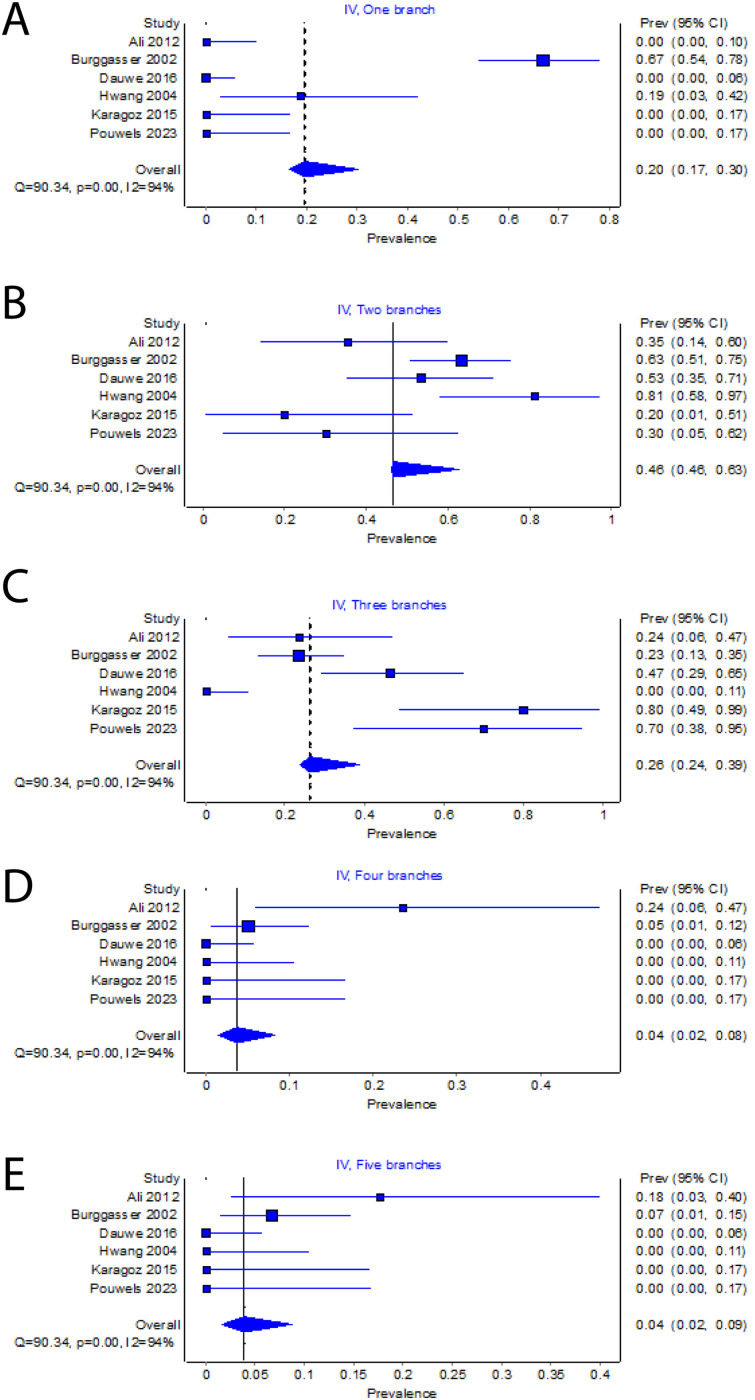
Forest plot of the variation in deep temporal nerve (DTN) branches.

A study by Geers et al. identified the innervation patterns of the anterior part of the TM, highlighting the deep-superficial distribution.^15^ Intramuscular dissection of the anterior DTN branches revealed that although some small rami exclusively supply the deep belly, larger branches penetrate deep muscle fibres to innervate deep and superficial bellies. Chang et al. delineated the superior-inferior distribution of innervation within the TM^9^ (Figure 2). The inferior part of the muscle comprises anterior and posterior regions, with the anterior region receiving innervation from the buccal nerve's superior branch forming an intramuscular plexus for the superior aspect and longer branches for the inferior aspect, while the posterior region is innervated by the masseteric nerve's anterior and posterior branches. In the superior part, divided into anterior, middle and posterior regions, the anterior region is supplied by the superficial and deep branches of the anterior DTN, arising from the superior branch of the buccal nerve, while the middle region is innervated by the middle DTN branching from the mandibular nerve and the posterior region by the posterior DTN originating from mandibular nerve or as a branch of the masseteric nerve.

### Variation in TM innervation

**Figure 3:**
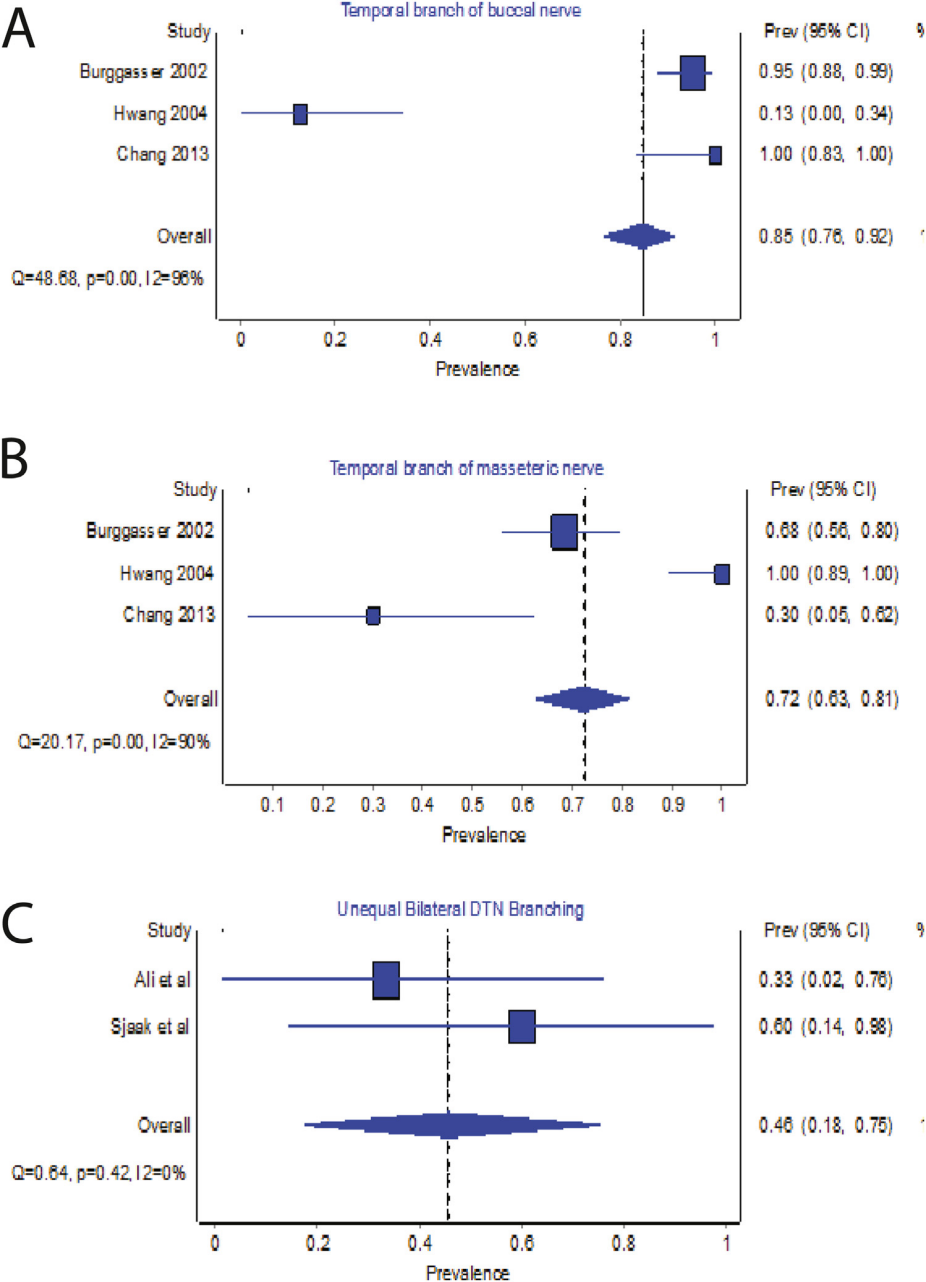
Forest plot of (a) temporal branch of the buccal nerve, (b) temporal branch of the masseteric nerve and (c) Unequal bilateral deep temporal nerve (DTN) branching.

Based on the findings of 143 specimens, the most prevalent variance of DTN was the two branches, with a pooled prevalence established at 46% (95% CI: 46%-63%, I^2^=94%), followed by three branches (26%, 95% CI:24%-39%) and one branch (20%, 95% CI: 17%-30%) (Figure 3). Only 4% of specimens had four or five branches. In a subgroup analysis of 86 patients, 85% (95% CI: 76%-92%, I^2^=98%) and 72% (95% CI: 63%-81%, I^2^=90%) were found to be innervated by the temporal branch of the buccal nerve and temporal branch of the masseteric nerve, respectively (Figure 3a and 3b). Additionally, peripheral anastomoses were observed between the three nerves in 12% of the specimen in the study by Burggasser et al.^10^ The findings by Wong (2022) indicated a predominant pattern, with the DTN originating alongside the inferior alveolar nerve from the mandibular nerve in most cases, while a smaller proportion originated from the maxillary nerve.^17^ A subgroup analysis of 2 studies (n=11) demonstrated 46% (95% CI:18%-75%, I^2^=0%) unequal bilateral DTN branching^1^^,^^20^ (Figure 3c).

## Discussion

**Figure 4:**
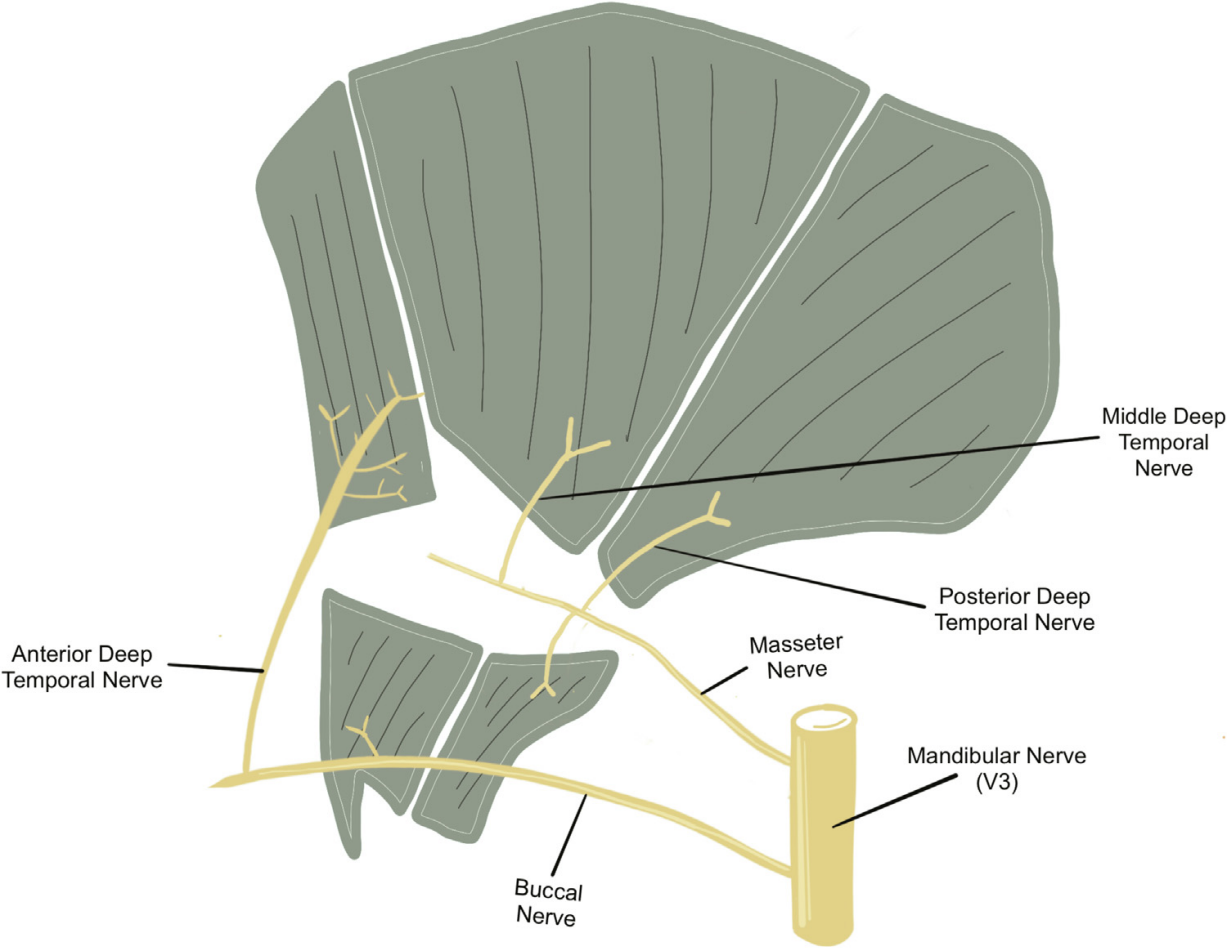
Nerve supply to the temporalis muscle.

Known variations in the anatomy of temporalis innervation have been documented in anatomical studies over the past three decades. However, to date, this systematic review and meta-analysis represent the first attempt to summarise the existing evidence regarding this topic. Our findings revealed an evolving understanding of the distribution of temporalis innervation. The most recent update by Chang et al. delineated the temporalis into five regions based on the innervation patterns^9^ (Figure 4). Additionally, significant variations in the DTN and temporal branches of the buccal and masseteric nerves were observed. Moreover, disparities between DTN branches on the left and right sides have been noted, prompting further investigation. However, it is important to acknowledge that the quality of evidence regarding these variations remains poor.

Identifying specific functional compartments is vital for achieving selective activation. The neuromuscular partitioning pattern may facilitate the harvesting of segmental muscle flaps while minimising the denervation of adjacent functional compartments within the TM.^9^ This approach holds the potential to reduce donor-site hollowing and enhances clinical outcomes in smile restoration. Furthermore, it could enable selective neurectomy as a non-invasive, effective and convenient procedure.^21^ A thorough understanding of the precise anatomical distribution of nerves and innervation pattern of the resected nerve are essential. For example, we hypothesise that hypertrophy of the anterocentral part of the temporal muscle can be reduced without compromising the masticatory function if the anterior division of the DTN is selectively severed.^22^

DTN transfer, often involving the anterior DTN, is frequently used for direct muscle neurotisation, nerve transfer and babysitter procedures in selective blinking restoration.^18^^,^^23^ Recent studies have also underscored the importance of middle deep temporal branches.^4^^,^^23^ Notably, the middle division exhibits the most anatomical consistency, greatest length and a more posterior location, indicating a lesser impact on the mastication force.^19^ However, there remains a lack of clarity in the nomenclature distinguishing the middle and posterior branches. The discrepancy between studies in identifying the maximum number of branches and naming the most consistent branches raises questions about the need for standardisation in nomenclature. Moreover, the relationship between the number of branches in DTN(s) and improved neural redundancy for the temporalis after nerve transfer procedures remains uncertain in the current literature. Another crucial consideration is whether nerve preservation, known for yielding better clinical outcomes, extends to the temporalis flap and whether these clinical outcomes are correlated with the number of branches.^24^

This review is subject to certain limitations. Anatomical cadaveric research typically faces constraints due to the small sample sizes. Studies with differing results from non-indexed literature in the selected databases may have been excluded, and there is a possibility that the most sensitive and specific search regarding the topic was not conducted. Additionally, individual assessments by the authors during the article selection process may have increased the likelihood of excluding potential cases that were not reported in the scientific community, particularly from countries outside of Asia and Europe. The lack of clarity in nomenclature and inadequately detailed data necessitated interpretation of certain results. The studies included were also limited to cadaveric studies that employed anatomical dissection methods. In future studies, electro-neuro-stimulation could be employed to map nerve distribution and branches in patients, offering a non-invasive method that could facilitate the recruitment of a larger number of participants.^25^^,^^26^

Finally, it is difficult to correlate the anatomical findings of this systematic review with clinical consequences. Based on the findings of our review, we postulate that the innervation of the front, middle and dorsal part of the TM might differ. Second, we hypothesise that after surgeries such as temporalis myoplasty, denser innervation of the muscle and consequently more stimuli might induce a more spontaneous smile. Theoretically, temporalis myoplasty could be combined with crossover nerve graft; however, technical surgical aspects and also the occurrence of synkinesis should be accounted for. Moreover, patient-related factors and surgical techniques should be considered.

## Conclusion

In conclusion, this systematic review and meta-analysis have provided valuable insights into the variations in temporalis innervation, marking a significant step forward in our understanding of this complex anatomical aspect. The findings underscore the evolving nature of our knowledge in this field. Moreover, the observed differences in the branches of the DTN and temporal branches of the buccal and masseteric nerves, as well as the noted variances between DTN branches on the left and right sides, emphasise the intricacies involved. However, it is essential to recognise the limitations inherent in the current body of evidence, which predominantly consists of anatomical studies. Moving forward, further research endeavours are warranted to enhance the quality and depth of our understanding, ultimately facilitating advancements in clinical practice and education within the realm of temporalis innervation.

## Author contributions

**Initial idea:** SP

**Literature search and data analysis:** ZYW, KQO

**Writing the article:** ZYW, KQO, KI, NvH, SP

**Final approval:** ZYW, KQO, KI, NvH, SP
